# Supplementary material for: Structural basis of fast N-type inactivation in Kv channels
Source: Nature. 2025 Aug 6;645(8082):1081–9. doi: 10.1038/s41586-025-09339-7 (PMC12460158; doi:10.1038/s41586-025-09339-7)
Supplement: Supplementary file 1 — Supplementary Figures 1–3 [file 41586_2025_9339_MOESM1_ESM.pdf]

nature portfolio

https://doi.org/10.1038/s41586-025-09339-7

Supplementary information

Structural basis of fast N-type inactivation in K_v channels

In the format provided by the authors and unedited

Supplementary information

Structural basis of fast N-type inactivation in Kv channels

Xiao-Feng Tan [1,4*], Ana I. Fernández-Mariño [1,3,4*], Yan Li [2], Tsg-Hui Chang [1] and Kenton J. Swartz [1*]

Affiliations:

1 Molecular Physiology and Biophysics Section, Porter Neuroscience Research Center, National Institute of Neurological Disorders and Stroke, National Institutes of Health, Bethesda, MD 20892

2 NINDS Proteomics Core Facility, Porter Neuroscience Research Center, National Institute of Neurological Disorders and Stroke, National Institutes of Health, Bethesda, MD 20892

3 Department of Physiology and Biophysics, University of Colorado, Anschutz Medical Campus, Aurora, CO 80045

4 These authors contributed equally.

* Address correspondence to: xiaofeng.tan@nih.gov

ana.fernandez-marino@cuanschutz.edu

swartzk@ninds.nih.gov

Table of Contents

Page 3-4 **Supplementary Figure 1|** Data processing workflow for the cryo-EM structure of the C-terminal mVenus tagged Shaker Kv channel.

Page 5-6 **Supplementary Figure 2|** Data processing workflow for the cryo-EM structure of the full-length Shaker Kv channel with enhanced inactivation mutations.

Page 7-8 **Supplementary Figure 3|** Data processing workflow for the cryo-EM structure of the full-length Shaker Kv channel with enhanced inactivation mutations in the presence of additional free N-terminal peptide.

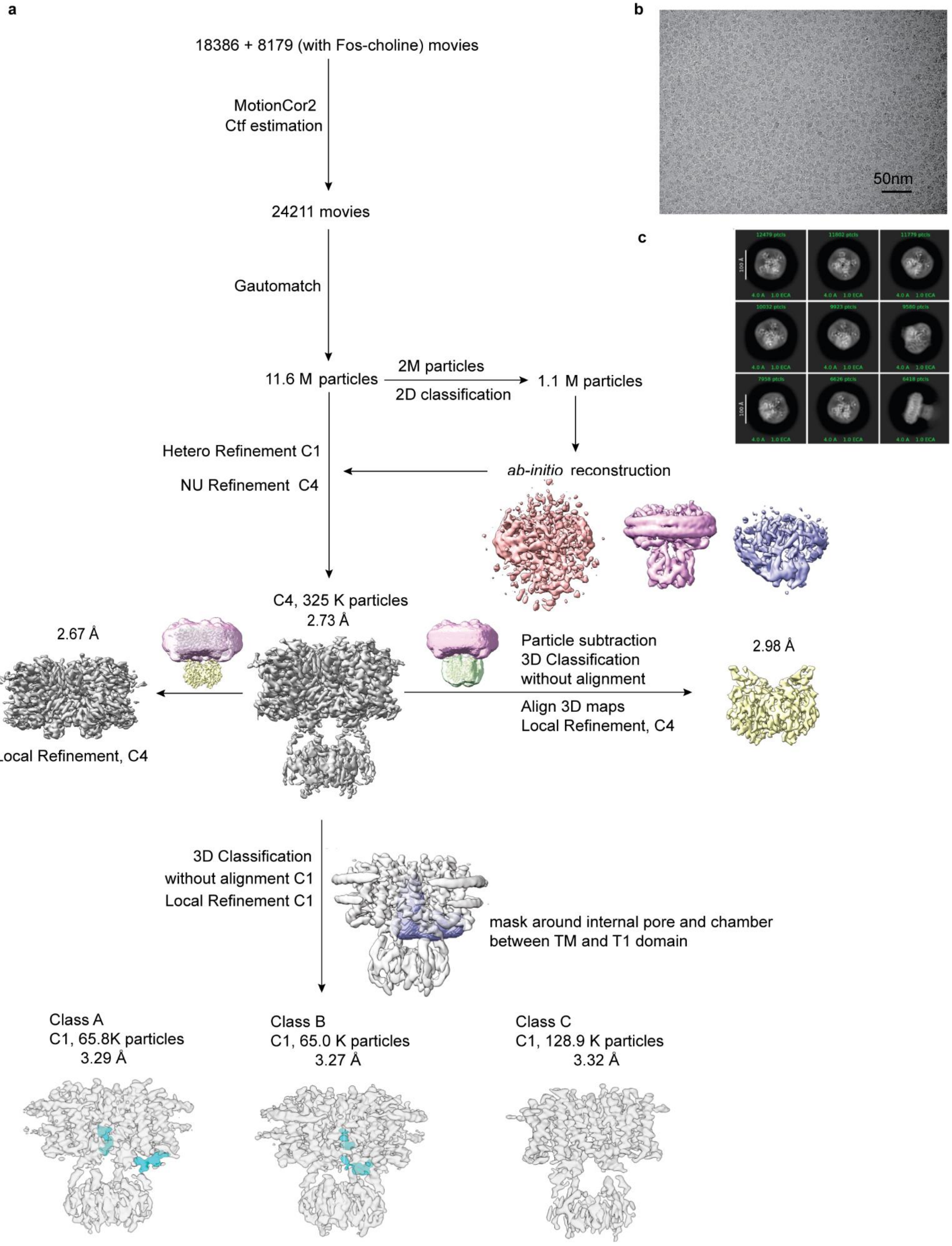
a
18386 + 8179 (with Fos-choline) movies
MotionCor2
Ctf estimation
24211 movies
Gautomatch
11.6 M particles
2M particles
2D classification
1.1 M particles
Hetero Refinement C1
NU Refinement C4
ab-initio reconstruction
C4, 325 K particles
2.73 Å
2.67 Å
Local Refinement, C4
Particle subtraction
3D Classification
without alignment
Align 3D maps
Local Refinement, C4
2.98 Å
3D Classification
without alignment C1
Local Refinement C1
mask around internal pore and chamber
between TM and T1 domain
Class A
C1, 65.8K particles
3.29 Å
Class B
C1, 65.0 K particles
3.27 Å
Class C
C1, 128.9 K particles
3.32 Å
b
50nm
c

← Supplementary Figure 1| Data processing workflow for the cryo-EM structure of the C-terminal mVenus tagged Shaker Kv channel.

a) Cyro-EM data processing pipeline for the C-terminal mVenus tagged Shaker Kv channel. b) Representative micrograph from the 8179 movies collected in Fos-choline. Typically about 5% of micrographs are discarded and at least 50% are of comparable quality to the representative one shown. c) 2D class averages of particles showing different orientations.

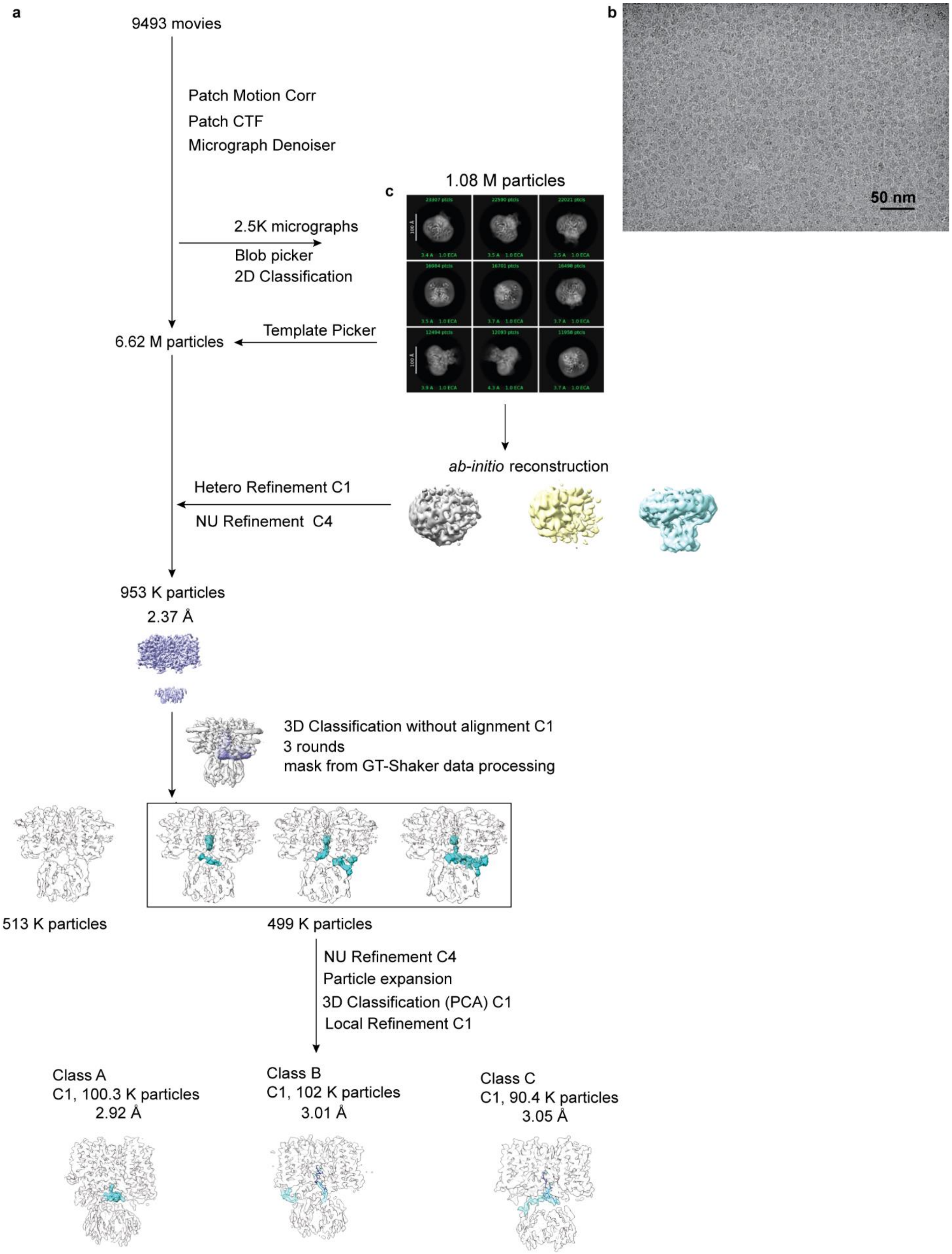
a
9493 movies
Patch Motion Corr
Patch CTF
Micrograph Denoiser
2.5K micrographs
Blob picker
2D Classification
6.62 M particles
Template Picker
Hetero Refinement C1
NU Refinement C4
953 K particles
2.37 Å
3D Classification without alignment C1
3 rounds
mask from GT-Shaker data processing
513 K particles
499 K particles
NU Refinement C4
Particle expansion
3D Classification (PCA) C1
Local Refinement C1
Class A
C1, 100.3 K particles
2.92 Å
Class B
C1, 102 K particles
3.01 Å
Class C
C1, 90.4 K particles
3.05 Å
b
50 nm
c
1.08 M particles
ab-initio reconstruction

← **Supplementary Figure 2|** Data processing workflow for the cryo-EM structure of the full-length Shaker Kv channel with enhanced inactivation mutations.

a) Cyro-EM data processing pipeline for the full-length Shaker Kv channel with enhanced inactivation mutations. **b**) Representative micrograph from the 9493 movies collected. Typically about 5% of micrographs are discarded and at least 50% are of comparable quality to the representative one shown. **c**) 2D class averages of particles showing different orientations.

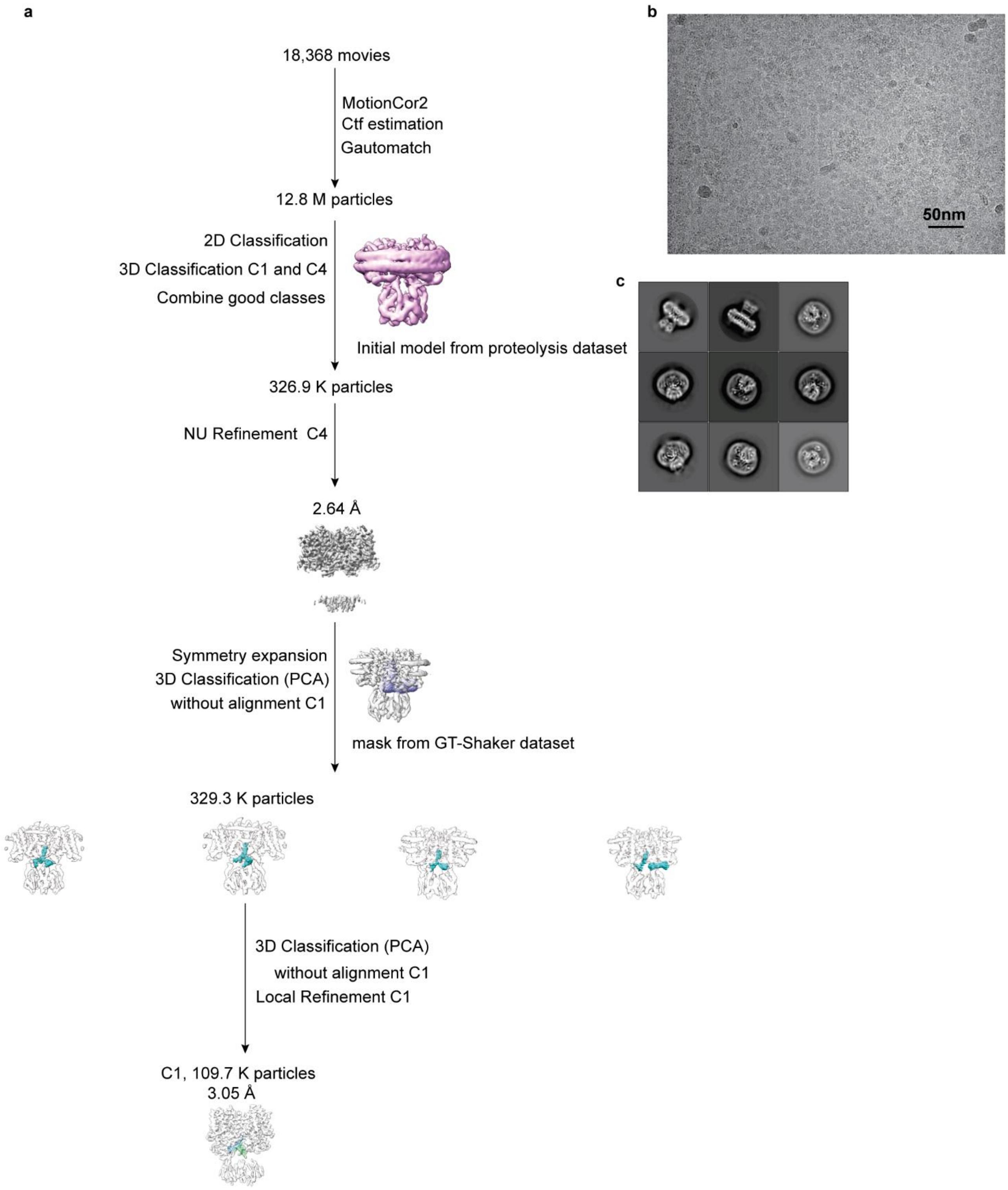
a
18,368 movies
MotionCor2
Ctf estimation
Gautomatch
12.8 M particles
2D Classification
3D Classification C1 and C4
Combine good classes
Initial model from proteolysis dataset
326.9 K particles
NU Refinement C4
2.64 Å
Symmetry expansion
3D Classification (PCA)
without alignment C1
mask from GT-Shaker dataset
329.3 K particles
3D Classification (PCA)
without alignment C1
Local Refinement C1
C1, 109.7 K particles
3.05 Å
b
50nm
c

← **Supplementary Figure 3|** Data processing workflow for the cryo-EM structure of the full-length Shaker Kv channel with enhanced inactivation mutations in the presence of additional free N-terminal peptide.

a) Cyro-EM data processing pipeline for the full-length Shaker Kv channel with enhanced inactivation mutations in the presence of additional free N-terminal peptide. **b**) Representative micrograph from the 18,368 movies collected. Typically about 5% of micrographs are discarded and at least 50% are of comparable quality to the representative one shown. **c**) 2D class averages of particles showing different orientations.
